# The Moderating Role of Age on Cognition Among Old Cancer Survivors: Comparison With Older Adults Without Cancer

**DOI:** 10.1093/geroni/igaa057.484

**Published:** 2020-12-16

**Authors:** Kun Wang, Hee Yun Lee, Jessica Neese

**Affiliations:** 1 University of Alabama, Tuscaloosa, Alabama, United States; 2 MSW, Tuscaloosa, Alabama, United States

## Abstract

Considering that 64% of cancer survivors are older adults, it is plausible that the overlap of cancer and old age may worsen older cancer survivors’ cognitive functioning. The current study aimed to test the effect of cancer history on cognition in comparison with older adults without cancer history and examine how age groups moderated the association between cancer and cognition. A subsample of 9197 participants drawn from the Health and Retirement Study (HRS) Wave13 were included in this cross-sectional study. Total word recall (score range: 0-20), mental status (0-15), and total cognition (0-35) were the three continuous dependent variables. Multilinear regressions were conducted with and without the interaction term (cancer history * age group). Findings showed cancer survivors tended to be older, Non-Hispanic white males, and higher educational attainment. Cancer history (B= .14, p< .05) was significant only for mental status. Cancer survivors had higher mental status scores than older adults without cancer history. The moderation effects of age groups on total word recall (p< .05), mental status (p< .01) and total cognition (p< .01) were significant only in the 85+ group. After adjusting for other variables, cancer survivors in the 85+ age group had significantly higher mental status and total cognition scores than their non-cancer counterparts. Posttraumatic growth can potentially explain the surprising result that older cancer survivors had higher cognition scores. Another possible explanation is that high cognitive functioning lengthened survival, and not cancer increased cognition. More experimental studies are needed to explore the current study’s findings further.

